# Use of Genetic Algorithms for Design an FPGA-Integrated Acoustic Camera

**DOI:** 10.3390/s22082851

**Published:** 2022-04-08

**Authors:** Sanja Grubeša, Jasna Stamać, Mia Suhanek, Antonio Petošić

**Affiliations:** 1 Department of Electroacoustics, Faculty of Electrical Engineering and Computing, University of Zagreb, 10000 Zagreb, Croatia; mia.suhanek@fer.hr (M.S.); antonio.petosic@fer.hr (A.P.); 2 Geolux d.o.o., Ljudevita Gaja 62, 10430 Samobor, Croatia; geolux@geolux.hr

**Keywords:** broadband acoustic camera, MEMS microphones, beamforming, genetic algorithm, FPGA SoC

## Abstract

The goal of this paper is to design a broadband acoustic camera using micro-electromechanical system (MEMS) microphones. The paper describes how an optimization of the microphone array has been carried out. Furthermore, the final goal of the described optimization is that the gain in the desired direction and the attenuation of side lobes is maximized at a frequency up to 4 kHz. Throughout the research, various shapes of microphone arrays and their directivity patterns have been considered and analyzed using newly developed algorithms implemented in Matlab. A hemisphere algorithm, genetic algorithm, and genetic square algorithm were used to find the optimal position and number of microphones placed on an acoustic camera. The proposed acoustic camera design uses a large number of microphones for high directional selectivity, while a field programmable gate array system on a chip (FPGA SoC) is selected as the processing element of the system. According to the obtained results, three different acoustic camera prototypes were developed. This paper presents simulations of their characteristics, compares the obtained measurements, and discusses the positive and negative sides of each acoustic camera prototype.

## 1. Introduction

Global insecurity and different threats to state borders are currently increasing, while the security of private properties is significantly diminished due to the availability of new complex technology to human smugglers and various criminal and terrorist organizations [1,2]. Therefore, there is a serious need for innovative solutions which are more resistant to interference, with more reliable threat detection in the field of border protection. Standard existing solutions are, in most cases, based on the combination of a radar detector with video camera surveillance in the optical and thermal spectrum [3,4]. In addition, vibrational and seismic detectors and touch barriers can be added for systems that demand an even higher detection reliability [5]. Variations and combinations of the mentioned systems often depend on the configuration of the protected object, area, terrain configuration and vegetation. It can be observed that the combination of several systems gives a more reliable detection and a lower probability of interfering with the system [6].

According to research [7,8], acoustic systems developed so far do not give adequate results, and are rarely used in real scenarios. There are only a few developed systems which use a more advanced signal processing and multi-microphone signal correlation [9]. However, even these systems have several major disadvantages, such as relatively high sensitivity to all sounds within a certain environment, low and unreliable ability to localize sounds in an environment, a very short detection range, and poor classification with a high number of false detections, which is a consequence of all of the above mentioned.

Bearing in mind the aforementioned, the aim of the research described in this article is to find an innovative acoustic threat detection solution which would be suitable for installation in different security systems. This type of solution would improve performance and detection, and furthermore disable the interference and sabotage of the system. The primary idea is to develop an acoustic detector based on the correlation of sound obtained from a large number of microphones (i.e., acoustic camera). This type of correlation principle could precisely locate sound in space, and thus create a map of amplitude and frequency distribution in space and time. Combining a large number of microphones would enhance the detection range and improve localization and classification, i.e., it would significantly increase the probability of threat detection while reducing the number of false alarms. In addition, this type of sensor would be highly compatible for combining it with video and radar detectors due to its working principle (i.e., it can perfectly complement the existing detectors in the most difficult working conditions). In many cases, low-power FMCW radar sensors struggle when trying to detect targets within any environment with denser vegetation [10]. Since it is almost impossible to move through such an environment without creating a sound, acoustic sensors would be a very good asset to radars. On the other hand, in open spaces where movement without making noticeable sounds is easier, radars are excellent in detection, and thus the whole system would be significantly more reliable in all conditions. In order to interfere with such combined systems, one would have to intrude with at least two sensors. In the case of a combination with a video system, three sensors which rely on completely different physical principles would be a necessity. It can be concluded that such an approach significantly complicates the interference and increases the likelihood that the target or threat would still be detected by at least one of the detection systems.

Acoustic cameras present a relatively new approach and only a few finished products are commercially available. All available products are designed for application in noise measurements and the acoustic characterization of different noise sources with small spatial spacing [11,12,13,14]. Therefore, it can be concluded that these acoustic cameras are primarily developed for laboratory or controlled conditions, i.e., long outdoor exposure and use without proper protection (i.e., adequate enclosure) could damage the microphones and reduce or completely disable their performance.

**Figure 1:**
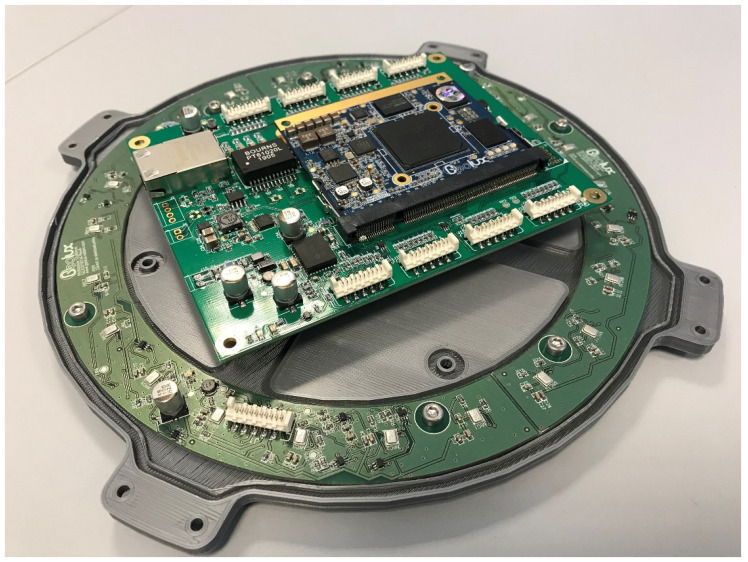
Acoustic camera motherboard module with mounted electronic components.

Keeping in mind our primary goal, which is to design a broadband acoustic camera using micro-electromechanical system (MEMS) microphones [15,16,17,18], an optimization of the microphone array has been performed in a way that the gain in the desired direction and attenuation of side lobes is maximized at a frequency up to 4 kHz. Increasing the frequency at which the acoustic camera design was accomplished, increases the cutoff frequency, i.e., the maximum frequency for which the camera can be used [19]. In our previous research [19,20], several simulations were performed considering square, circular and hemisphere-shaped MEMS arrays with varying numbers of microphones and varying spacing between the microphones. Since the directivity pattern of a microphone array is frequency dependent [21], simulations were performed in the frequency band from 63 Hz to 8 kHz, though the results are presented only for three frequencies of interest, i.e., 1 kHz, 2 kHz and 4 kHz. The proposed acoustic camera design uses a large number of microphones for high directional selectivity while a field programmable gate array system on a chip (FPGA SoC) was selected as the processing element of the system, which is shown in Figure 1. SoC FPGAs are semiconductor devices which integrate programmable logic with hard processor cores. Therefore, FPGA SoC enables the easy acceleration of processing intensive algorithms applied to a large number of signals from all the microphones in the FPGA array, and enables easy interfacing from FPGA to advanced reduced instruction set computer machine (ARM) processors in the chip. Signal processing algorithms implemented in FPGA are designed modularly to enable simple parallelization and parametrization of the number of microphones. Intermediate results from the FPGA processing algorithms are combined for all microphones and transferred into the synchronous dynamic random access memory (SDRAM). ARM processors in the SoC then use those results for the final steps of processing, with a lower demand on the processing power. The final steps of the algorithm and visualization are implemented in the ARM processing system to speed up development and enable easier changes in the algorithm and visualization [22,23,24].

In order to achieve our goal, which is to design a robust yet small acoustic camera which can be used in different security systems, various shapes of microphone arrays and their directivity patterns have been considered and analyzed using newly developed algorithms implemented in Matlab. In particular, the hemisphere algorithm, genetic algorithm and genetic square algorithm [25,26] have been used to find the optimal position and number of microphones placed on an acoustic camera. The genetic algorithm (GA) has been used for the hemisphere-shaped acoustic camera, while the genetic square algorithm has been used for a plate-shaped acoustic camera. According to the obtained results, three prototypes of the acoustic camera were developed. This paper presents simulations of their characteristics, compares the obtained measurements, and discusses the positive and negative sides of each acoustic camera prototype.

## 2. The Acoustic Camera Designs

In this research, the following three prototypes were developed:

- A prototype of a hemisphere-shaped acoustic camera—*Acoustic Camera 1*;
- A prototype of a plate-shaped acoustic camera (i.e., in the form of a plate consisting of four plates measuring 20 × 20 cm with a uniform distribution of microphone arrays)—*Acoustic Camera 2*, and
- A prototype of a plate-shaped acoustic camera (i.e., in the form of a plate consisting of four optimal acoustic plates with 24 microphones)—*Acoustic Camera 3*.

### 2.1. A Hemisphere-Shaped Acoustic Camera—Acoustic Camera 1

**Figure 2:**
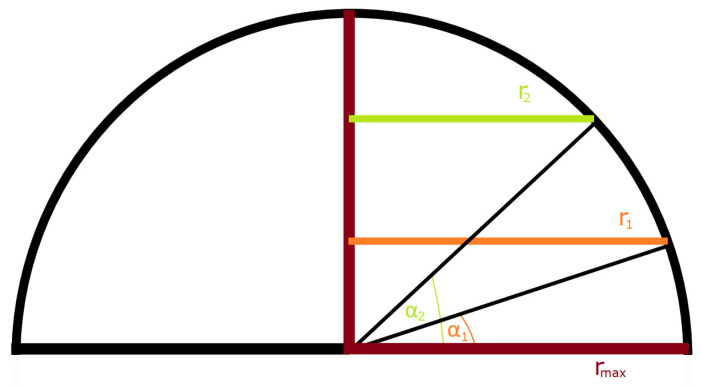
A hemisphere with a microphone on top and two microphone arrays arranged in circles with radii r_1_ and r_2_.

The first simulation was carried out for the hemisphere-shaped acoustic camera with one microphone on top and microphone arrays arranged in two circles located randomly (in the first iteration) on the hemisphere which is shown in Figure 2.

Using the hemisphere algorithm, the exact location of the mentioned circles is calculated in a way to obtain the maximum gain in the main lobe at a frequency of f = 1 kHz, i.e., *Acoustic camera 1* is optimized for a frequency of 1 kHz. The final aim and purpose of the acoustic camera is to successfully detect drones, and therefore the frequencies of interest are 1 kHz, 2 kHz and 4 kHz. In the calculations, the defined input parameters were the radius of the hemisphere (r_max_ = 0.20 m), the distance d between the microphones in the circles (less than half of the wavelength for the frequency f = 1 kHz (λ/2 = 0.1715 m)), the distance between the circular microphone arrays on a hemisphere (Δα = α_2_ − α_1_ greater or equal to 15°), and the total number of microphones in an array (maximum 150). Furthermore, the parameter α_1_ determines the radius r_1_ and the height at which the first circle is located, while n_1_ is the number of microphones in the first circular array. Following the same analogy, α_2_ determines the radius r_2_ and the height of the second circle, while n_2_ is the number of microphones in the second circular array. The parameter G represents the main lobe gain (0° in the directional characteristic), while the parameter A represents the attenuation of side lobes. The total number of microphones *n* is the sum of the number of microphones in both circles and the one microphone on the top of the hemisphere, i.e., *n* = *n*_1_ + *n*_2_ + 1. Table 1 shows the parameters obtained using this type of optimization.

**Figure 3:**
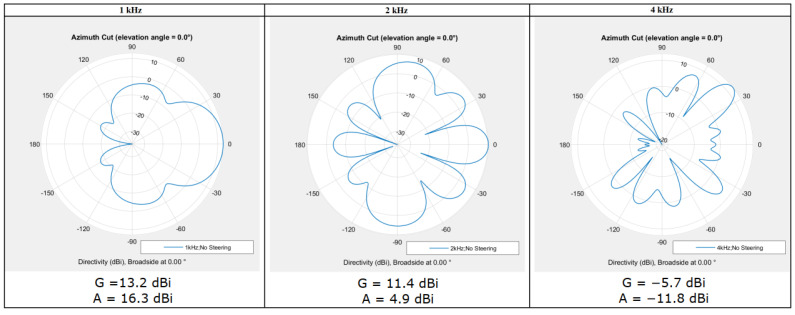
Directional characteristics for 1, 2 and 4 kHz (optimization carried out for f = 1 kHz).

For *Acoustic camera 1* optimized for a frequency of 1 kHz, directional characteristics and values of the main lobe gain, and attenuation of side lobes for 1, 2 and 4 kHz frequencies are calculated. The calculations and the obtained results are shown in Figure 3.

When observing and analyzing the results obtained through optimization for the frequency of 1 kHz, it can be concluded that the obtained acoustic camera is not broadband, and that it is necessary to optimize the number and arrangement of microphones on the hemisphere for all considered frequencies. The next step would be adding one or more microphone circles to the hemisphere using the genetic algorithm to determine the number and arrangement of microphones on the acoustic camera. Furthermore, as previously mentioned, it is necessary to increase the frequency while designing the broadband acoustic camera (from 1 kHz to 4 kHz).

#### 2.1.1. Implementation of Genetic Algorithm (GA) in Case of *Acoustic Camera 1*

The genetic algorithm (GA) is an adaptive empirical search algorithm based on the evolutionary ideas of natural selection and genetics [26]. Genetic algorithms are commonly used to generate high-quality solutions to optimization and search problems by relying on biologically inspired operators such as mutation, crossover and selection.

In particular, the optimization was carried out using the genetic algorithm implemented in Matlab for all three frequencies (1 kHz, 2 kHz and 4 kHz). In order to achieve the overall evaluation of efficiency of *Acoustic camera 1*, and a large signal gain at 1, 2 and 4 kHz (i.e., to obtain a broadband camera), two scoring functions were introduced. For each unit, i.e., each acoustic camera from the population, the genetic algorithm calculates the main lobe gain G and the attenuation of side lobes A for all three frequencies (1 kHz, 2 kHz and 4 kHz). Using these parameters (gain G and attenuation A) for each specified frequency, a calculation is performed according to Equation (1) for SC_1_, where the values G_0_ and A_0_ are the desired target values obtained by optimization.

SC_1_ = CA(1 + G/G_0_) + CB(1 + A/A_0_), (1)

In the developed model, the ratio between CA and CB equals 4 which means that the main lobe gain G has four times higher weight than the attenuation of side lobes A. The values G_0_ and A_0_ are the desired target values obtained by optimization, and for the developed model, these values are G_0_ = 20 dBi and A_0_ = 30 dBi, respectively. The maximum value of the first scoring function SC_1_ is 22.3.

The second scoring function SC calculates the “overall score” of *Acoustic Camera 1* at all frequencies, provided that the parameters obtained at each frequency have the same weight:

SC = SC_1_(1 kHz) + SC_1_(2 kHz) + SC_1_(4 kHz), (2)

As a result of SC_1_ having the same weight at each frequency, the maximum value for the overall score SC is 66.9.

This particular genetic algorithm postulates an initial population of 40 acoustic cameras which are created in the first iteration. To be more precise, the algorithm creates 40 randomly selected acoustic cameras in the shape of a hemisphere with a radius of r = 0.2 m. Furthermore, the number of circles which will be arranged on the camera is randomly selected, and this number can be between 1 and 19 (due to the condition that the distance between the circles must be higher than or equal to 5°). The algorithm nevertheless examines the microphone distance in circles which must be larger than 0.015 m. After creating the initial population, each following population consists of x best acoustic cameras from the previous iteration, y new ones obtained by crossover and z new randomly created hemisphere-shaped acoustic cameras. Before each new iteration, the hemisphere-shaped acoustic cameras from the current iteration are sorted from the highest total score to the lowest. After that step, x acoustic cameras (with the best total score) are transferred to the next iteration, and the hemisphere-shaped acoustic camera with the highest score is utilized using crossover to obtain y new hemisphere-shaped acoustic cameras. The crossover is done between the hemisphere-shaped acoustic camera with the highest score and each subsequent hemisphere. Thus, a new hemisphere-shaped camera is created with microphones in position of every other microphone from the best hemisphere and every microphone from the subsequent hemisphere that meets the condition that the distance from all other microphones is greater or equal to 0.015 m. The limit of the total microphone number is predefined when using crossover (i.e., the maximum is 150 microphones). If the crossover process creates multiple hemisphere-shaped acoustic cameras with the same number and arrangement of microphones, these duplicates are replaced by new random hemisphere-shaped acoustic cameras. In addition to the x hemisphere-shaped acoustic cameras with the best score and y hemisphere-shaped acoustic cameras created by the crossover process, the next iteration includes z new hemisphere-shaped acoustic cameras which are created in the same way as the initial population, i.e., randomly. Finally, 750 iterations are performed for each population. Table 2 shows all created populations.

To clarify Table 2, the population marked 2 was created from the four hemisphere-shaped acoustic cameras with the best score from the previous iteration, 12 hemisphere-shaped acoustic cameras obtained using crossover, and 24 brand new randomly generated hemisphere-shaped acoustic cameras. The population marked 5 is the same as population 2, however this population allows a smaller distance between circles, i.e., the distance between circles can be higher than or equal to 1°. As previously mentioned, 750 iterations are carried out for five created populations. In addition, the maximum score in each population is determined and finally a hemisphere-shaped acoustic camera is established in each population that can achieve such a score. In order to compare and discuss the results in a more comprehensive way after all the performed simulations, the results are sorted from the highest total score to the lowest, which is shown in Table 3.

Table 4 presents the parameters for the best hemisphere-shaped acoustic cameras from each population. To clarify the table, the position of each circle on the acoustic camera is determined by the angle in degrees, and the number of microphones in the circle (at the height determined by that particular angle) is written in brackets behind each angle.

#### 2.1.2. Developing a Prototype of a Hemisphere-Shaped Acoustic Camera—*Acoustic Camera 1*

**Figure 4:**
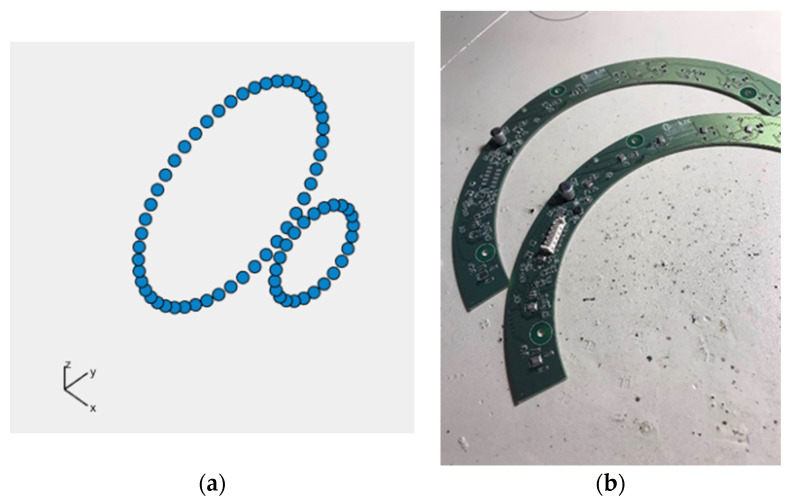
(**a**) Positions of microphone array circles on the prototype of a hemisphere-shaped acoustic camera in coordinate system, x being the broadside direction (*Acoustic Camera 1*); (**b**) Printed circuit board with mounted electronic components for the prototype of a hemisphere-shaped acoustic camera (*Acoustic Camera 1*).

**Figure 5:**
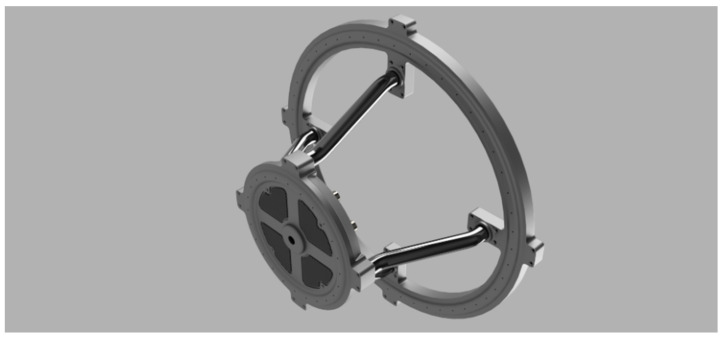
The designed prototype of a hemisphere-shaped acoustic camera (*Acoustic Camera 1*).

Based on all conducted simulations, a decision was made that the prototype of a hemisphere-shaped acoustic camera will have a radius r = 0.2 m with two microphone arrays arranged in circles whose radii and heights are determined by angles 0° and 65°. The described design is shown in Figure 4. Figure 5 displays the actual prototype of a hemisphere-shaped acoustic camera.

### 2.2. Plate-Shaped Acoustic Cameras—Acoustic Camera 2 and Acoustic Camera 3

**Figure 6:**
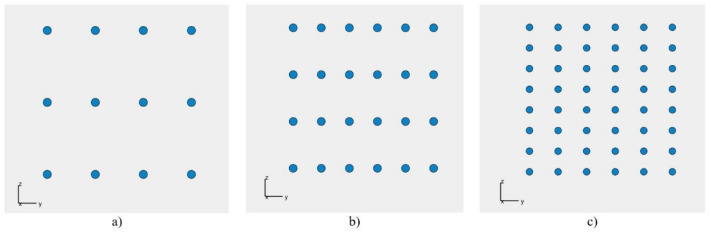
Square array in coordinate system, x being the broadside direction, with (**a**) 12 microphones; (**b**) 24 microphones; (**c**) 48 microphones.

The base of the plate-shaped acoustic camera is a 20 cm long and 20 cm wide panel, on which 12, then 24, and finally 48 uniformly arranged microphones are placed, which is shown in Figure 6. This is carried out using Matlab R2021b (MathWorks Inc., Natick, MA, USA) and the Sensor Array Analyzer which is a part of the Matlab Phased Array System Toolbox (Matlab R2021b). Instead of using the genetic algorithm in this particular iteration of research, the experimental method was utilized. Using this method, it was determined that the best results were obtained for a uniform distribution of microphones on a flat plate, provided that the minimum distance between the microphones was greater than 0.015 m. Furthermore, the same condition was later used in the genetic algorithm.

Furthermore, the values of main lobe gain G and side lobe attenuation A are calculated for all three microphone layouts on the plate-shaped acoustic camera. The calculated values are presented in Table 5. For the 1 kHz frequency, the side lobe attenuation is marked with N/A (not applicable) because the directivity patterns for all three arrays are in the shape of a cardioid, i.e., there are no side lobes.

To enable the efficiency evaluation of the square microphone arrays, and the possibility of comparison with the previously described *Acoustic Camera 1*, it is necessary to apply already introduced and described scoring functions. The calculated values of the first scoring function SC_1_ and the total scoring function SC for square arrays with 12, 24 and 48 microphones are presented in Table 6.

From results presented in Table 5 and Table 6, it can be concluded that the best result was obtained for the square array with 24 microphones.

#### 2.2.1. Implementation of Genetic Algorithm (GA) in Case of Plate-Shaped Acoustic Cameras

**Figure 7:**
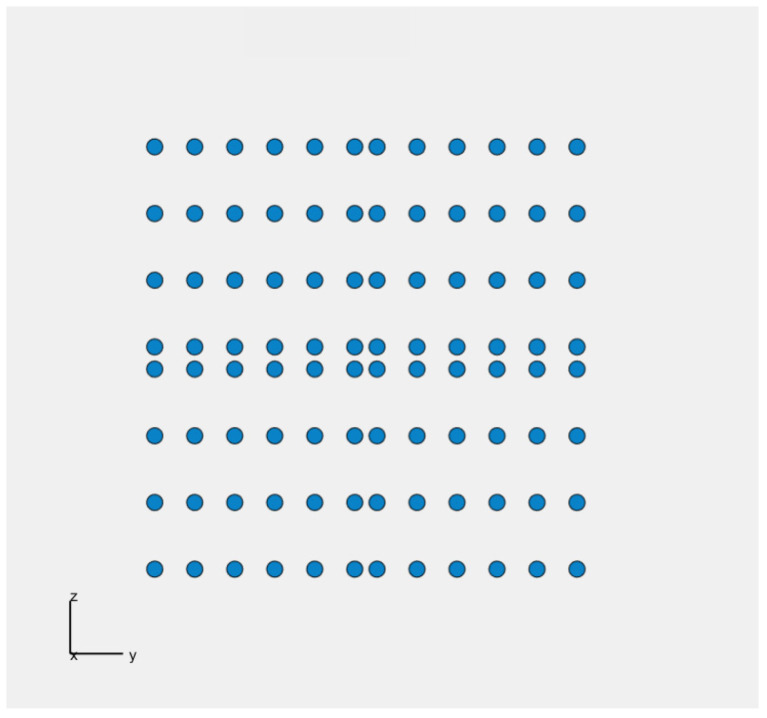
The positions of the microphones on the plate-shaped acoustic camera in coordinate system, x being the broadside direction —*Acoustic Camera 2*.

The genetic algorithm named GA_square is developed and implemented in Matlab to obtain optimal results for the plate-shaped acoustic camera. Once again, the first step is to create an initial population P1 consisting of 40 acoustic cameras. Each acoustic camera consists of four panels (dimensions 20 × 20 cm) arranged in such a way that the second panel is the axial symmetry of the first panel with respect to the *x*-axis, the third panel is the axial symmetry of the first panel with respect to the *y*-axis, and the fourth panel is the central symmetry of the first panel with respect to the origin. Keeping in mind that the best result is achieved for a uniform arrangement of 24 microphones placed on the panel, in this case, for each panel, the maximum number of microphones is 24, i.e., the total number of microphones on the *Acoustic Camera 2* is 96 (shown in Figure 7).

**Figure 8:**
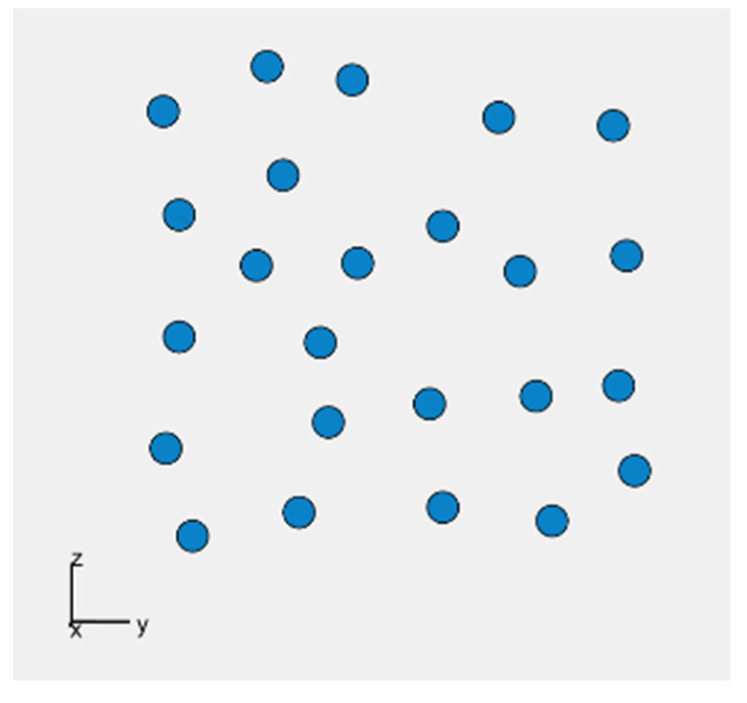
The optimal panel with 24 microphones obtained using the GA_square algorithm in coordinate system, x being the broadside direction.

**Figure 9:**
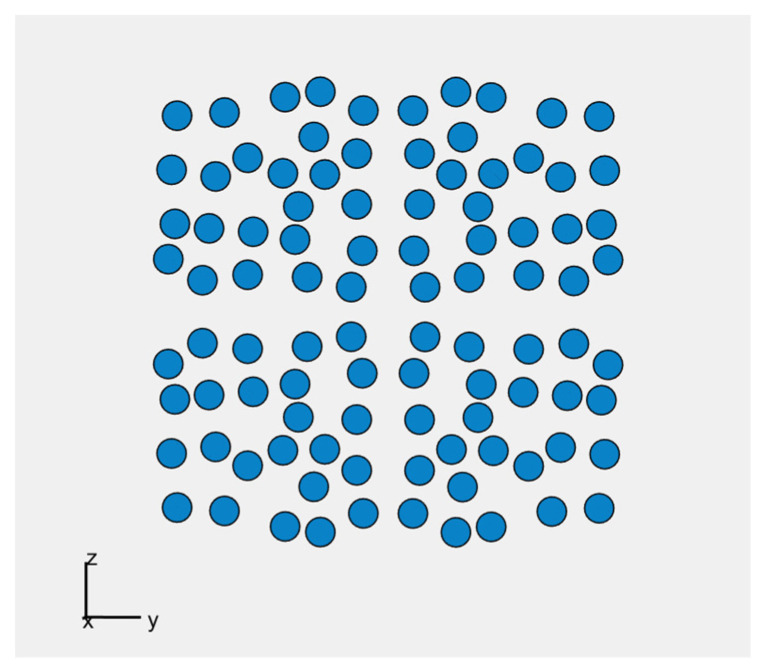
An acoustic camera in coordinate system, x being the broadside direction, consisting of four optimal panels from Figure 8 arranged in such a way that the second panel is the axial symmetry of the first panel with respect to the x-axis, the third panel is the axial symmetry of the first panel with respect to the y-axis, and the fourth panel is the central symmetry of the first panel with respect to the origin—*Acoustic Camera 3*.

The minimum number of microphones on each panel is predefined and equals 20. For each version of *Acoustic Camera 3*, the algorithm randomly selects a microphone number between 20 and 24 and places them on one panel in a way that they satisfy the condition that the distances between all microphones are greater than or equal to 0.015 m. After that, three more identical panels are created and fitted together with the first one to form a camera with dimensions 40 × 40 cm as previously described using axial and central symmetry. Scoring functions SC_1_ (1 kHz), SC_1_ (2 kHz), SC_1_ (4 kHz) and the total scoring function SC are calculated for each created acoustic camera in the initial population. In addition, the obtained acoustic cameras are sorted from the highest to the lowest total score (SC). The next iteration includes the four cameras with the best score, and 12 acoustic cameras created by crossover. Furthermore, 24 acoustic cameras are created in the same way as the initial population to keep the initially selected population size in each iteration. The crossover is carried out after sorting the acoustic cameras, i.e., between one panel of the acoustic camera with the highest total score and one panel of every subsequent acoustic camera. Using crossover, new acoustic camera panels are created with microphones in positions of every other microphone from the highest rated acoustic camera panel, and any microphone that satisfies the distance condition (d ≥ 0.015 m) from the lower rated acoustic camera panels. This procedure continues until all microphones from the lower rated acoustic camera panel are used, or until the total number of microphones reaches the maximum microphone number. For each panel obtained using crossover, three more identical panels are created. Finally, these four panels are arranged in the aforementioned way, and together form a new acoustic camera which is created using the crossover process. After 750 iterations, an optimal acoustic camera with 96 microphones was obtained, i.e., an acoustic camera with 24 microphones on each acoustic camera panel. Figure 8 shows the optimal panel with 24 microphones obtained by this algorithm, while Figure 9 shows *Acoustic Camera 3* consisting of four panels from Figure 8 arranged in the aforementioned way.

#### 2.2.2. Developing the Prototypes of Plate-Shaped Acoustic Cameras—*Acoustic Camera 2* and *Acoustic Camera 3*

**Figure 10:**
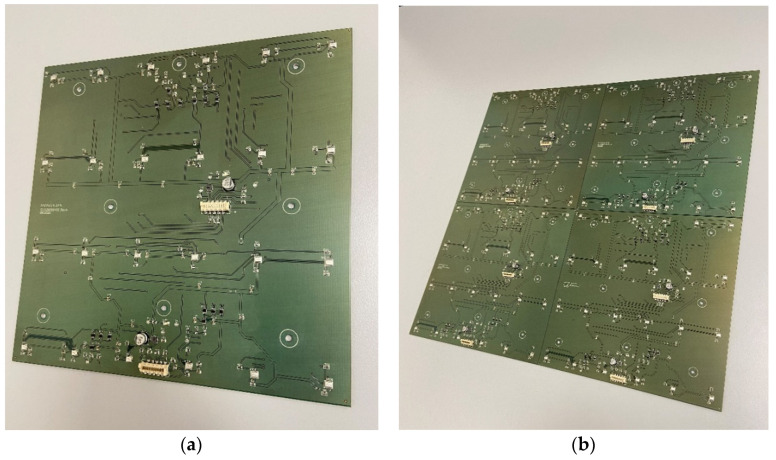
(**a**) A single printed circuit board with mounted electronic components for the prototype of plate-shaped acoustic camera; (**b**) Four boards arranged together to form *Acoustic Camera 2*.

In order to develop a prototype of a plate-shaped acoustic camera with the same dimensions as the prototype of a hemisphere-shaped acoustic camera, it is necessary to use four panels (dimensions 20 × 20 cm) and assemble them together to create an acoustic camera with final dimensions of 40 × 40 cm. The results from Table 6 show that the panel with 24 uniformly arranged microphones has achieved the highest rating. Therefore, the first prototype of a plate-shaped acoustic camera consists of four such panels and represents *Acoustic Camera 2* as shown in Figure 7. The printed circuit board with mounted electronic components for the prototype of a plate-shaped acoustic camera—*Acoustic Camera 2*—is shown in Figure 10.

**Figure 11:**
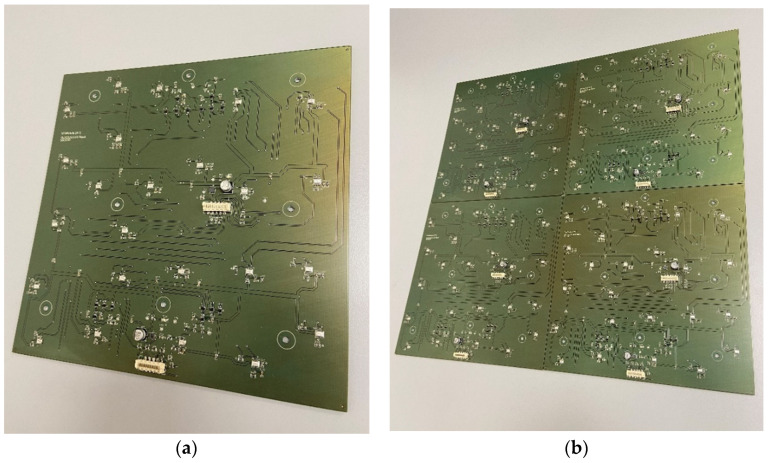
(**a**) A single printed circuit board with mounted electronic components for the prototype of plate-shaped acoustic camera; (**b**) Four boards arranged together to form *Acoustic Camera 3*.

Furthermore, based on all the conducted simulations, a decision was made that the second plate-shaped prototype acoustic camera will have the final dimensions of 40 × 40 cm and will consist of four panels shown in Figure 8. The described design represents the final prototype of *Acoustic Camera 3* as shown in Figure 9. The printed circuit board with mounted electronic components for the prototype of a plate-shaped acoustic camera—*Acoustic Camera 3*—is shown in Figure 11.

## 3. The Comparison of Different Acoustic Camera Prototypes

*Acoustic Camera 1* has the lowest number of microphones (i.e., 72 microphones) when compared to *Acoustic Camera 2* and *Acoustic Camera 3*, which both have 96 microphones. When considering the camera shape, the *Acoustic Camera 1* prototype, as well as its enclosure, is the most complex. When observing Table 7, *Acoustic Camera 2* has the highest gain at all frequencies, while *Acoustic Camera 3* has the highest side lobe attenuation.

### 3.1. The Simulation Results of Acoustic Camera Prototypes

**Figure 12:**
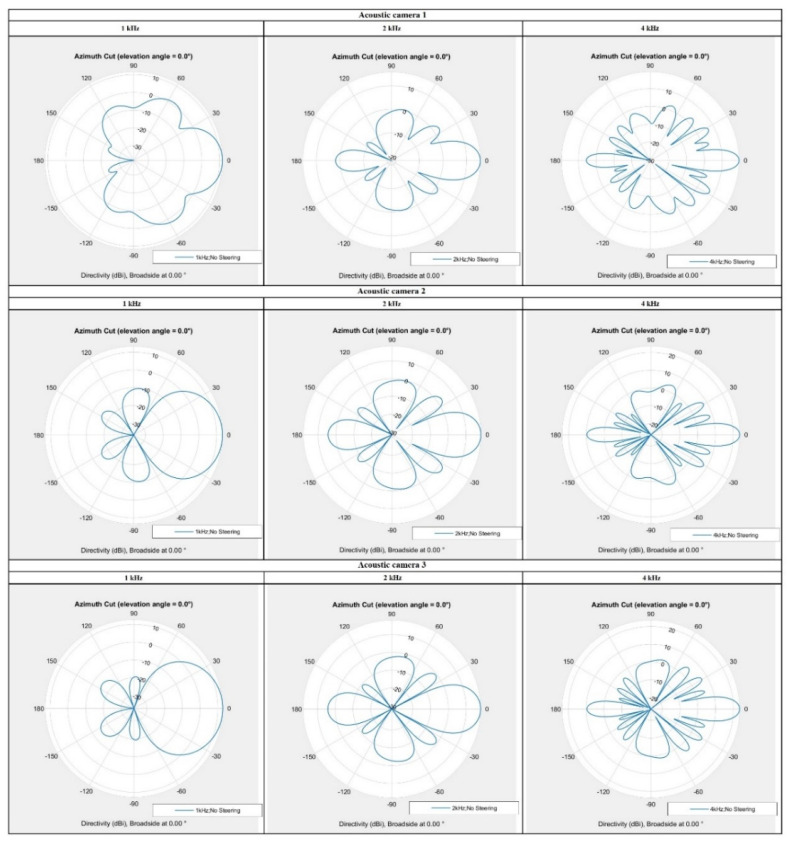
Directional characteristics of all acoustic camera prototypes (at frequencies 1, 2 and 4 kHz).

The directional characteristics of the three prototypes are shown in Figure 12, while Table 7 shows the main lobe gain and side lobe attenuation values at frequencies 1, 2, and 4 kHz for all three acoustic camera prototypes. For all the prototypes at all desired frequencies, significant gain in the main lobe and attenuation of the side lobes have been achieved, and thus three broadband acoustic cameras were obtained. Although the results are presented only for the three frequencies of interest, detailed simulations were made in the frequency bands from 63 Hz up to 8 kHz (i.e., in the one-third octave frequency bands) and, as expected, all three prototypes (up to the frequency of 2000 Hz) have a directional characteristic of the cardioid, and, at frequencies higher than 2000 Hz, the main lobe gain G is greater than 10 and the attenuation of side lobe A is greater than 7.

The total score obtained from simulations for all three camera prototypes is shown in Table 8.

From the results presented in Figure 12 and Table 7 it can be observed that each prototype is a broadband acoustic camera, however, Table 8 shows that the highest overall score has been achieved with *Acoustic Camera 3*, and therefore it can be concluded that the best broadband acoustic camera prototype consists of four optimized panels with 24 microphones arranged in the same way as described in Section 2.1.1.

### 3.2. The Measurement Results of Acoustic Camera Prototypes

**Figure 13:**
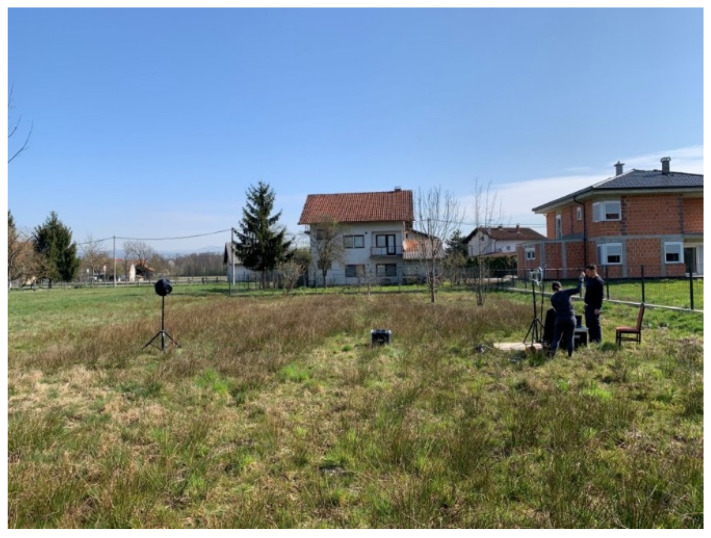
The acoustic camera prototype placement during the measurement.

To determine the directional characteristic uniformity of the acoustic camera prototype, the following measurement is carried out. The center of the acoustic camera was placed at a height of z_c_ = 1.55 m, while the omnidirectional sound source was placed at a height of z = 1.55 m and at a distance of 8 m from the acoustic camera prototype (see Figure 13). Since the beamforming of the acoustic camera prototype is done exclusively using software, it is important that the acoustic camera has a uniform directional characteristic, [27,28,29]. In order to test the directivity function, broadband signal (pink noise) was used to observe the response over the whole frequency range, not just for a few selected frequencies. During the measurement, the air temperature was 15.1 °C, the humidity was 30%, and the wind speed was 0.2 m/s in the direction from the loudspeaker sphere to the acoustic camera. In addition to those measurements, sound levels were also measured using a Brüel & Kjær 2250 (Brüel & Kjær, Nærum, Denmark) sound level meter. The sound level meter measured the sound pressure L_Zeq_ in dB, with an integration time of 10 s.

**Figure 14:**
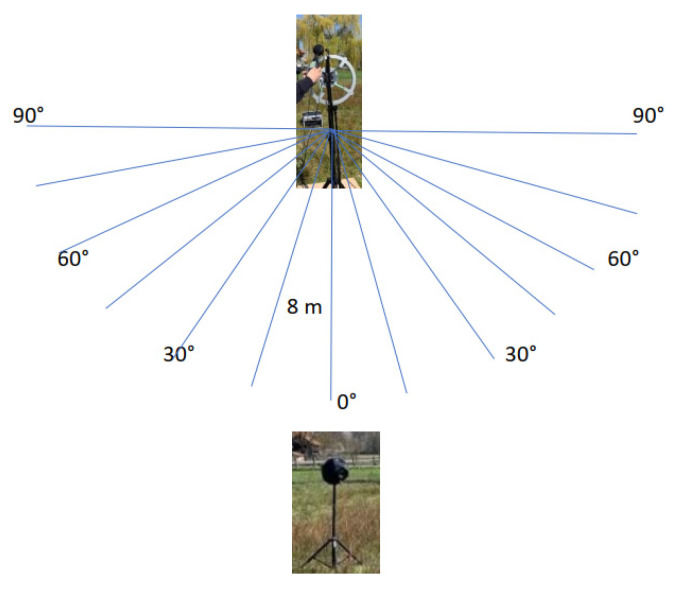
The rotation (by degrees) of omnidirectional sound source (i.e., sphere speaker) in front of the acoustic camera prototype.

The omnidirectional sound source, i.e., the loudspeaker sphere, was rotated by different degrees in front of the acoustic camera prototype, maintaining the same distance of 8 m as shown in Figure 14. The chosen steps were 15 degrees to the left and right up to a maximum of 90 degrees. Table 9 shows the measured sound pressure values using the Brüel & Kjær 2250 sound meter and acoustic camera prototype obtained from the previously described measurement setup.

**Figure 15:**
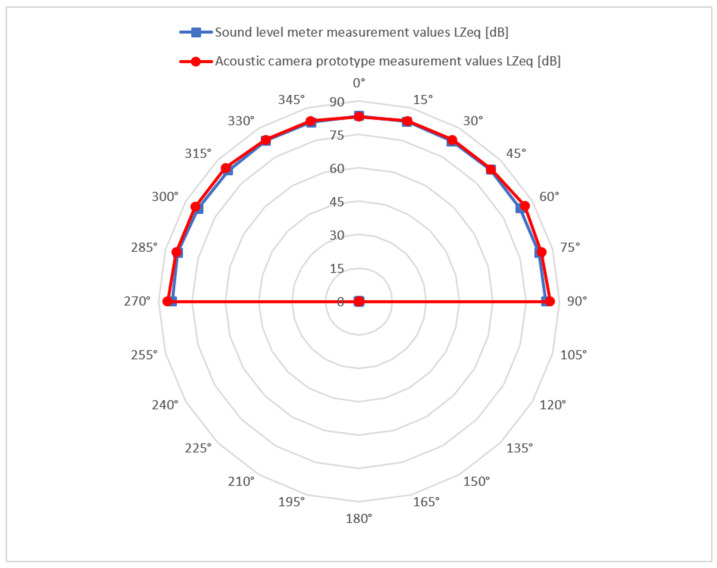
The comparison of the measured directional characteristic of the acoustic camera prototype (red colour) with the measured values obtained with the Brüel & Kjær 2250 sound meter (blue colour).

**Figure 16:**
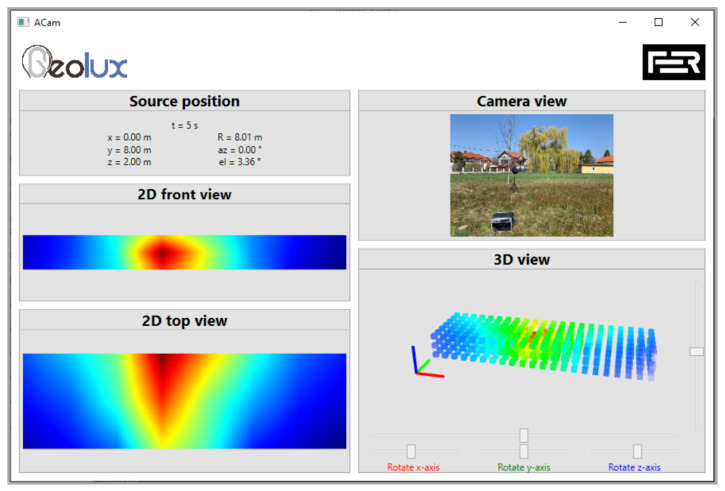
Measurements obtained with acoustic camera prototype.

The comparison of the measured directional characteristic of the acoustic camera prototype with the measured values obtained with the Brüel & Kjær 2250 sound meter is shown in Figure 15. It can be noticed that the directional characteristic of the acoustic camera prototype is uniform. Figure 16 shows the measurement results gained by the acoustic camera prototype, when the source is located directly in front of the camera (0°) at a distance of 8 m.

## 4. Conclusions

The primary idea of the described research in this paper (and project KK.01.2.1.01.0103 4D Acoustical Camera) is to develop an acoustic detector based on the correlation of sound obtained from a large number of micro-electromechanical system (MEMS) microphones (i.e., acoustic camera). The motivation for the acoustic camera development is that the type of correlation principle which the acoustic camera uses could precisely locate sound in space, and thus create a map of amplitude and frequency distribution in space and time. Furthermore, combining a large number of microphones would increase the detection range, improving the precision of localization and classification, i.e., it would significantly increase the probability of threat detection while reducing the number of false alarms.

Keeping in mind the described goal, the paper describes and explains an optimization of the microphone array, which has been carried out. The required optimization outcome is that the gain in the desired direction and the attenuation of side lobes is maximized at a frequency up to 4 kHz, which has been achieved. The proposed acoustic camera design uses a large number of microphones for high directional selectivity, while FPGA SoC was selected as the processing element of the system.

*Acoustic Camera 1* has the lowest number of microphones (i.e., 72 microphones) when compared to *Acoustic Camera 2* and *Acoustic Camera 3*, which both have 96 microphones. Regarding the camera shape, the *Acoustic Camera 1* prototype, as well as its enclosure, is the most complex. *Acoustic Camera 2* has the highest gain at all frequencies, while *Acoustic Camera 3* has the highest side lobe attenuation. All the acoustic camera prototypes have achieved significant gain in the main lobe and attenuation of the side lobes at all desired frequencies. Therefore, from all the results presented in the paper, it can be concluded that three broadband acoustic cameras were obtained. However, when observing Table 8 it can be noticed that the highest overall score was achieved with *Acoustic Camera 3*. Hence, it can be concluded that the best broadband acoustic camera prototype consists of four optimized panels with 24 microphones arranged in such a way that the second panel is the axial symmetry of the first panel with respect to the *x*-axis, the third panel is the axial symmetry of the first panel with respect to the y-axis, and the fourth panel is the central symmetry of the first panel with respect to the origin.

When comparing the measured directional characteristic of the acoustic camera prototype with the measured values obtained with the Brüel & Kjær 2250 sound meter, it can be noticed that the directional characteristic of the acoustic camera prototype is uniform.

All three of the discussed acoustic camera prototypes are already integrated into an existing Geolux low-power FMCW (frequency-modulated continuous wave) radar and video camera system.

After the development of three acoustic camera prototypes and all the associated algorithms, future work will be oriented toward the verification of the prototypes’ operation in real environments and conditions.

## Figures and Tables

**Table 1:** The calculated values obtained in optimization for a frequency f = 1 kHz.

r_max_	0.200 m
d	0.170 m
α_1_	20°
r_1_	0.188 m
*n*_1_	7
α_2_	35°
r_2_	0.164 m
*n*_2_	6
G	13.2 dBi
A	16.3 dBi
*n*	14

**Table 2:** Created populations using the genetic algorithm.

Population Mark	Population Size = x (Best from Previous Iteration) + y (New Ones Created Using Crossover) + z (New Ones Created Randomly)
1	40 = 12 + 12 + 16
2	40 = 4 + 12 + 24
3	40 = 4 + 4 + 32
4	40 = 2 + 12 + 26
5	40 = 4 + 12 + 24 STEP 1°

**Table 3:** Sorted results (according to the total score) for all created populations.

Population Mark	Population Size = x (Best from Previous Iteration) + y (New Ones Created Using Crossover) + z (Newly Created Randomly)	Score SC_1_ and the Overall Score SC
2	40 = 4 + 12 + 24	SC_1_: [15.2827 17.2282 18.6894] SC: 51.2003
4	40 = 2 + 12 + 26	SC_1_: [14.8595 17.3148 18.9160] SC: 51.0903
3	40 = 4 + 4 + 32	SC_1_: [16.8706 16.4777 17.7411] SC: 51.0894
1	40 = 12 + 12 + 16	SC_1_: [16.7452 16.7217 17.6133] SC: 51.0802
5	40 = 4 + 12 + 24 STEP 1°	SC_1_: [16.5735 16.4258 17.6911] SC: 50.6904

**Table 4:** The parameters for the best hemisphere-shaped acoustic cameras from each population.

Population Mark	The Circle Position on the Acoustic Camera [°] and the Number of Microphones (in Brackets)	The Total Number of Microphones on the Acoustic Camera
2	0° (48), 65° (24)	48 + 24 = 72
4	0° (45), 65° (15)	45 + 15 = 60
3	35° (33), 90° (1)	33 + 1 = 34
1	35° (30)	30
5	33° (35), 90° (1)	35 + 1 = 36

**Table 5:** Signal gain and attenuation for each square array version.

Frequency (Hz)	1000		2000		4000	
	G (dBi)	A (dBi)	G (dBi)	A (dBi)	G (dBi)	A (dBi)
Square array with 12 microphones	8.8709	N/A	13.8558	14.8744	14.3310	11.8358
Square array with 24 microphones	8.2234	N/A	13.5240	17.9620	18.5997	13.0778
Square array with 48 microphones	7.8124	N/A	13.1172	17.9620	18.5501	13.0778

**Table 6:** Obtained values of scoring functions for each version of the square array.

	SC_1_ (1 kHz)	SC_1_ (2 kHz)	SC_1_ (4 kHz)	SC
Square array with 12 microphones	13.5484	16.5340	16.5215	46.6038
Square array with 24 microphones	13.2894	16.6071	18.3117	48.2082
Square array with 48 microphones	13.1249	16.4444	18.2919	47.8612

**Table 7:** Signal gains and side lobe attenuation of each acoustic camera prototype.

Frequency (Hz)	1000		2000		4000	
	G (dBi)	A (dBi)	G (dBi)	A (dBi)	G (dBi)	A (dBi)
Acoustic Camera 1	11.6869	9.1196	15.8550	13.2938	19.5723	12.9075
Acoustic Camera 2	13.3476	23.6101	18.4138	15.7453	23.6812	15.2169
Acoustic Camera 3	12.7201	27.0348	18.1426	21.2696	23.3653	18.4144

**Table 8:** The overall score of all acoustic camera prototypes.

	SC_1_ (1 kHz)	SC_1_ (2 kHz)	SC_1_ (4 kHz)	SC
Acoustic Camera 1	15.2827	17.2282	18.6894	51.2003
Acoustic Camera 2	16.9130	18.4152	20.4869	55.8151
Acoustic Camera 3	16.8904	18.6750	20.5737	56.1391

**Table 9:** The measured sound pressure values using the Brüel & Kjær 2250 sound meter (keeping in mind the rotation in 15 degrees steps) and acoustic camera prototype.

Sound Source—Loudspeaker Sphere at a Distance R = 8 m—Pink Noise		
The Placement of Loudspeaker Sphere with Respect to the Sound Level Meter and Acoustic Camera	Sound Level Meter Measurement Values L_Zeq_ [dB]	Acoustic Camera Prototype Measurement Values L_Zeq_ [dB]
0°	83.4	83
15° left	83.4	84
30° left	83.5	84
45° left	83.3	85
60° left	83.5	85
75° left	84.4	85
90° left	84.0	86
15° right	83.6	84
30° right	83.2	84
45° right	83.8	84
60° right	83.8	86
75° right	83.8	85
90° right	84.0	86

## Data Availability

Not applicable.
